# Adipose-derived mesenchymal stem cells-derived exosome-mediated microRNA-342-5p protects endothelial cells against atherosclerosis

**DOI:** 10.18632/aging.102857

**Published:** 2020-02-24

**Authors:** Xiaohui Xing, Zhongchen Li, Xin Yang, Mengyou Li, Chao Liu, Yuejiu Pang, Liyong Zhang, Xueyuan Li, Guangcun Liu, Yilei Xiao

**Affiliations:** 1 Department of Neurosurgery, Shandong Provincial Qianfoshan Hospital, Shandong University, Jinan 250000, Shandong Province, P.R. China; 2 Department of Neurosurgery, Liaocheng People’s Hospital, Liaocheng 250000, Shandong Province, P.R. China; 3 Department of Neurosurgery, Shandong Provincial Hospital Affiliated to Shandong University, Jinan 250000, Shandong Province, P.R. China; 4 Department of Otolaryngology, General Hospital of Central Theater Command of PLA, Wuhan 430070, Hubei, China; 5 Department of Senile Neurology, Shandong Provincial Hospital Affiliated to Shandong University, Jinan 250000, Shandong Province, P.R. China

**Keywords:** miR-342-5p, adipose-derived mesenchymal stem cell, exosome, endothelial cells, atherosclerosis

## Abstract

Exosomes are reported to mediate several disease-related microRNAs (miRNAs) to affect the progression of diseases, including atherosclerosis. Here, we aimed to screen the atherosclerosis-associated miRNAs and preliminarily investigate the potential regulatory mechanism of atherosclerosis. First, the lesion model for human umbilical vein endothelial cells (HUVECs) was favorably constructed. Later, through RNA-sequencing and bioinformatics analyses, miR-342-5p was identified in lesion model for HUVECs. MiR-342-5p overexpression or knockdown evidently promoted or inhibited the apoptosis of HUVECs impaired by H_2_O_2_. Mechanistically, PPP1R12B was found to have great potential as a target of miR-342-5p in HUVECs impaired by H_2_O_2_, supported by RNA-sequencing and a series of bioinformatics analyses. Meanwhile, the effect of miR-342-5p on PPP1R12B expression in HUVECs’ lesion model was explored, revealing that miR-342-5p had an inhibitory role in PPP1R12B expression. Additionally, adipose-derived mesenchymal stem cells (ADSCs) in spindle-like shape and their derived exosomes with 30 to 150 nm diameter were characterized. Furthermore, results showed miR-342-5p was evidently decreased in the presence of ADSCs-derived exosomes. These findings indicated ADSCs-derived exosomes restrained the expression of miR-324-5p in lesion model. Collectively, this work demonstrates an atherosclerosis-associated miR-342-5p and reveals a preliminary possible mechanism in which miR-342-5p mediated by ADSCs-derived exosomes protects endothelial cells against atherosclerosis.

## INTRODUCTION

Atherosclerosis, commonly caused by the accumulation of lipid-laden macrophages in the arterial wall, is the major underlying contributor to cardiovascular diseases and brings much morbidity and mortality across the globe [1]. A series of hyperlipidemia-induced chronic inflammatory processes, covering complicated interactions among modified lipoproteins, monocytes and T lymphocytes with cellular components sourced from the vessel wall, facilitate the buildup of atherosclerosis lesion [2–4]. To date, numerous diagnostic techniques have been employed to assess the cardiovascular disease risk and definitive therapies, and been generally classified into invasive and noninvasive kinds. The former kind is exemplified by invasive angiography and optical coherence tomography, the latter kind is by blood biomarkers, stress testing, CT and nuclear scanning [1]. Of note, considerable studies concerning the cell and molecular biology of atherogenesis have enhanced the understanding of the underlying risk factors involved in atheroma development and its clinical features [5–8]. Therefore, continued research about the potential mechanism of atherogenesis is conductive to better combating this chronic disease.

Conveniently, the pathogenesis of atherosclerosis goes through three phases, including initiation, progression and complications [1]. Damage or dysfunction of endothelial cells are recognized as critical events in the initiation of atherosclerotic-plaque development [9]. The adaptation of arterial endothelial cells to disturbed blood flow is critical for, or even decisive in the susceptibility at branching sites of arteries, where atherosclerosis preferentially occurs [10]. It thus appears that seeking effective approaches to protect endothelial cells may provide fruitful strategies to prevent atherosclerosis progression.

MicroRNAs (miRNAs), a class of small and noncoding RNAs, are recognized as important post-transcriptional regulators of specific messenger RNAs (mRNAs) [11, 12]. Recently, a considerable literature has recognized the dysregulated expression of several miRNAs associated with pro-atherosclerosis and anti-atherosclerosis as the critical mechanisms of atherosclerosis development [13–15]. Numerous miRNAs are reported to exert essential regulated role in lipid metabolism, inflammation and atherogenesis, supported by the cases of miR-19b and miR-144-3p involved in regulation of lipid metabolism [16, 17], miR-92a and miR-155 as critical regulators of inflammation [18, 19], miR-30c and miR-126-5p preventing atherosclerosis [20, 21]. By this token, targeting miRNAs may potentially alleviate the development of atherosclerosis. Despite bountiful atherosclerosis-associated miRNAs reported, much remains to be done in seeking novel or valuable miRNAs and exploring their potential biological functions in atherosclerosis.

In this study, we aimed to screen the atherosclerosis-associated miRNAs and preliminarily investigate the potential regulatory mechanism of atherosclerosis. At first, RNA-sequencing was employed to comparatively analyze the differentially expressed miRNAs and mRNAs in patients with atherosclerosis and corresponding healthy controls. The lesion model for human umbilical vein endothelial cells (HUVECs) was constructed. Secondly, our aimed miRNA, miR-342-5p, was identified on basis of bioinformatics analysis and experimental verifications. The effect of miR-342-5p on the apoptosis of lesion model of HUVECs was also evaluated. According to a series of bioinformatics analysis and experimental confirmation, an atherosclerosis-associated target of miR-342-5p was identified. Finally, adipose-derived mesenchymal stem cells (ADSCs)-derived exosomes were characterized, and their effect on miR-342-5p expression in lesion model for HUVECs was investigated. Collectively, this work identified an atherosclerosis-associated miRNA (miR-342-5p) and unearthed a potential mechanism for this miRNA functioning in atherosclerosis.

## RESULTS

### Analyses of differentially expressed miRNAs and mRNAs by RNA-sequencing

**Figure 1:**
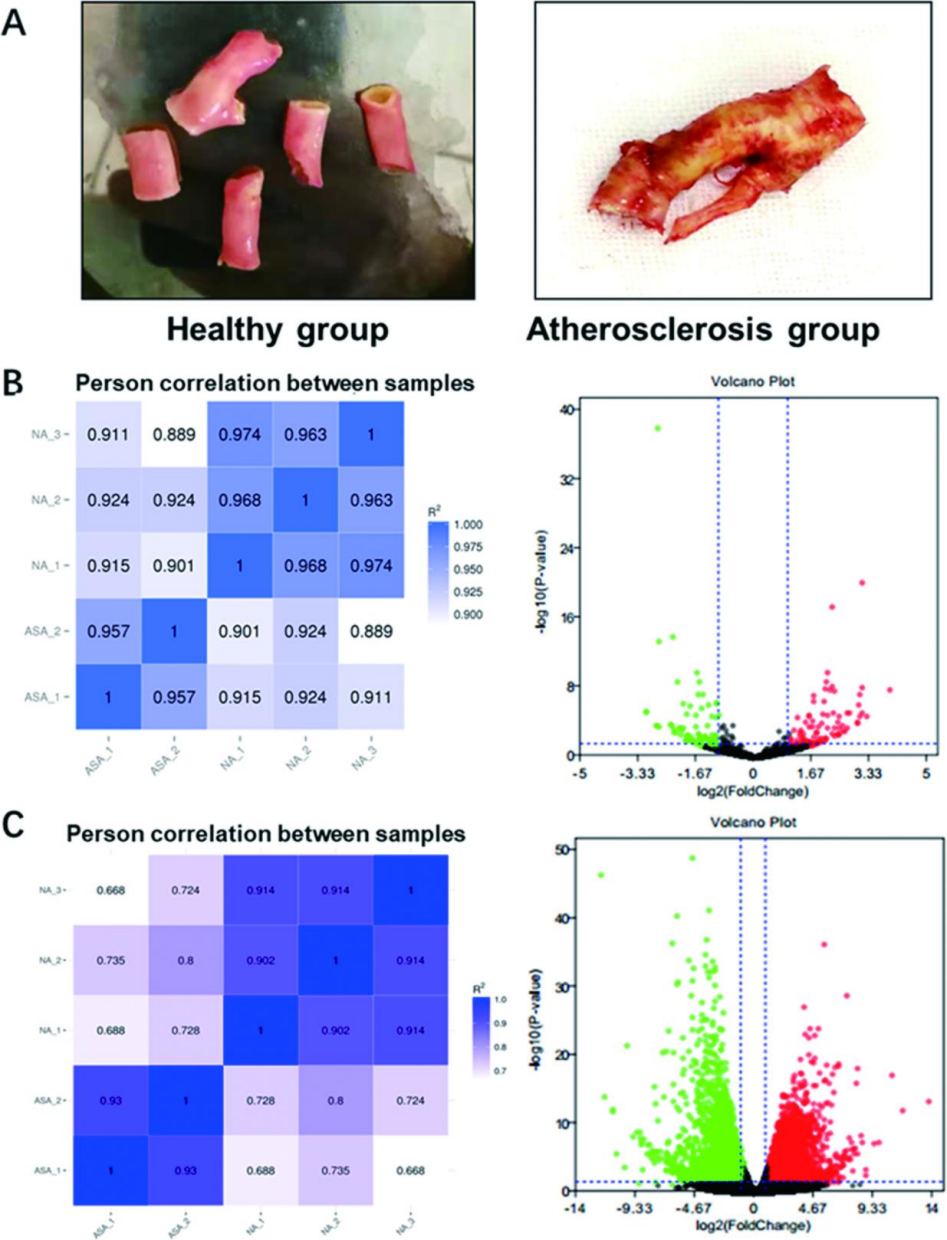
**Photos of collected samples and RNA-sequencing analysis of differentially expressed miRNAs and mRNAs in atherosclerosis.** (**A**) The normal carotid artery from patients with accident (Healthy group) and atherosclerotic plaque collected from patients with atherosclerosis (Atherosclerosis) were photo’d by cellphone. (**B**) The heat map (Left panel) and volcano plot (Right panel) of differentially expressed miRNAs and (**C**) mRNAs in atherosclerosis by RNA-sequencing. Red plots stand for upregulated genes and green ones represent downregulated genes with absolute log2FC>1 and *P* < 0.05. Black ones indicate those non-significant expressed miRNAs or mRNAs. miRNA, microRNA; mRNA, messenger RNA; FC, fold change.

For the purpose of analyzing differentially expressed miRNAs and mRNAs, three cases of atherosclerotic plaque collected from patients with atherosclerosis and two cases of normal carotid artery from patients with accident were separately used for RNA-sequencing. These samples were photo’d in Figure 1A, which the left panel presented the normal carotid artery (namely Healthy group) while the right panel showed the atherosclerotic plaque (namely Atherosclerosis group). Commonly acknowledged, biological replicates are usually required for any biological experiment, and usually defined as measurements of biologically distinct samples with biological diversity [22]. In general, the correlation coefficient closer to 1 stands for higher similarity on expression pattern between samples. Among RNA-sequencing process, biological replicates were conducted in our collected samples. The heat maps separately for miRNAs and mRNAs, clearly showing difference of intergroup samples and repetition of intra-group samples, were visualized in Figure 1B, 1C (Left panel). In RNA-sequencing test for screening differentially expressed miRNAs, the volcano plot was obtained with the criteria of |log_2_FC|>1 and *P* < 0.05. As shown in Figure 1B (Right panel), a total of 141 miRNAs were differentially expressed in atherosclerosis samples, wherein 68 were upregulated and 73 were downregulated. Similarly, the volcano plot was generated with the abovementioned criteria for RNA-sequencing test for screening differentially expressed mRNAs. As displayed in Figure 1C (Right panel), a total of 4,848 mRNAs were differentially expressed in atherosclerosis samples. Among them, 2,350 were upregulated and 2,694 were downregulated. Conjointly, these results indicate the reliability of RNA-sequencing and reveal differentially expressed miRNAs and mRNAs in atherosclerosis.

### The lesion model for HUVECs was constructed

**Figure 2:**
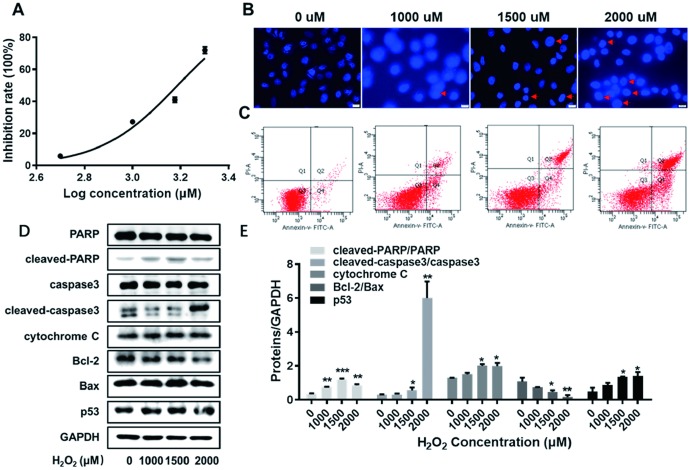
**The establishment of the lesion model for HUVECs.** (**A**) The inhibition rate of HUVECs exposed to H_2_O_2_ at varied concentrations (0, 500, 1000, 1500 and 2000 uM) were detected by CCK8 assay. (**B**) HUVECs exposed to H_2_O_2_ at varied concentrations (0, 1000, 1500 and 2000 uM) were visualized according to Hoechst staining kit assay. The red arrows indicate nuclear condensation or apoptosis. (**C**) The apoptosis rate of HUVECs exposed to H_2_O_2_ at varied concentrations (0, 1000, 1500 and 2000 uM) were detected by flow cytometry assay. (**D**) With the enhanced concentrations of H_2_O_2_ (0, 1000, 1500 and 2000 uM), the expression levels of cleaved-PARP/PARP, cleaved-caspase3/caspase3, cytochrome C, Bcl-2, Bax and p53 were detected by western blot assay. (**E**) The corresponding grey-scale maps of (**D**) were shown. *, *P*<0.05, **, *P*<0.01, ***, *P*<0.001. HUVECs, human umbilical vein endothelial cells; GAPDH, glyceraldehyde 3-phosphate dehydrogenase.

In order to establish the lesion model for endothelial cells, HUVECs were exposed to H_2_O_2_ at varied concentrations (0, 500, 1000, 1500 and 2000 uM). As shown in Figure 2A, the corresponding inhibition rates of HUVECs at varied concentrations were detected by CCK-8 assay. The IC_50_ of H_2_O_2_ to impair HUVECs was calculated as 1538 uM using Graphpad software. Thus, H_2_O_2_ at varied concentrations (0, 1000, 1500 and 2000 uM) was used in the following experiments. Next, HUVECs exposed to H_2_O_2_ at varied concentrations (0, 1000, 1500 and 2000 uM) were visualized according to Hoechst staining kit assay. As obviously observed in Figure 2B, the number of apoptotic body, indicated by red arrow, exerted an increase trend in HUVECs along with the increasing concentrations of H_2_O_2_. Later, the results obtained from flow cytometry assay showed that apoptosis rate of HUVECs was elevated along with the increasing concentrations of H_2_O_2_ (Figure 2C). This was evidence in the following summarized data in Table 1: 2.6%, 11.1%, 20.5% and 41.1% apoptosis rate of HUVECs at 0, 1000, 1500 and 2000 uM of H_2_O_2_, respectively. Furthermore, as analyzed by western blot assay, the expression levels of cleaved-PARP/PARP, cleaved-caspase3/caspase3, cytochrome C, and p53 were significantly arisen with the enhanced concentrations of H_2_O_2_ (Figure 2D, 2E, *P*<0.05, *P*<0.01, *P*<0.001). Meanwhile, the ratio of Bcl-2 to Bax demonstrated a descending trend as the concentrations of H_2_O_2_ aroused (Figure 2D, 2E, *P*<0.05, *P*<0.01). The abovementioned results collectively support the successful construction of the lesion model for HUVECs.

**Table 1:** The apoptotic rates of HUVECs exposed to H_2_O_2_ at varied concentrations.

**Apoptotic rate (%)**	**H_2_O_2_ concentration (μM)**			
	**0**	**1000**	**1500**	**2000**
**Early apoptosis in Q_2_ stage**	0.5	3.3	11.4	23.6
**Middle and advanced apoptosis in Q_4_ stage**	2.1	6.8	9.1	17.5
**Total**	2.6	11.1	20.5	41.1

Abbreviations: HUVECs, human umbilical vein endothelial cells.

### The aimed miRNA, miR-342-5p, is identified and promotes the apoptosis of HUVECs impaired by H_2_O_2_

**Figure 3:**
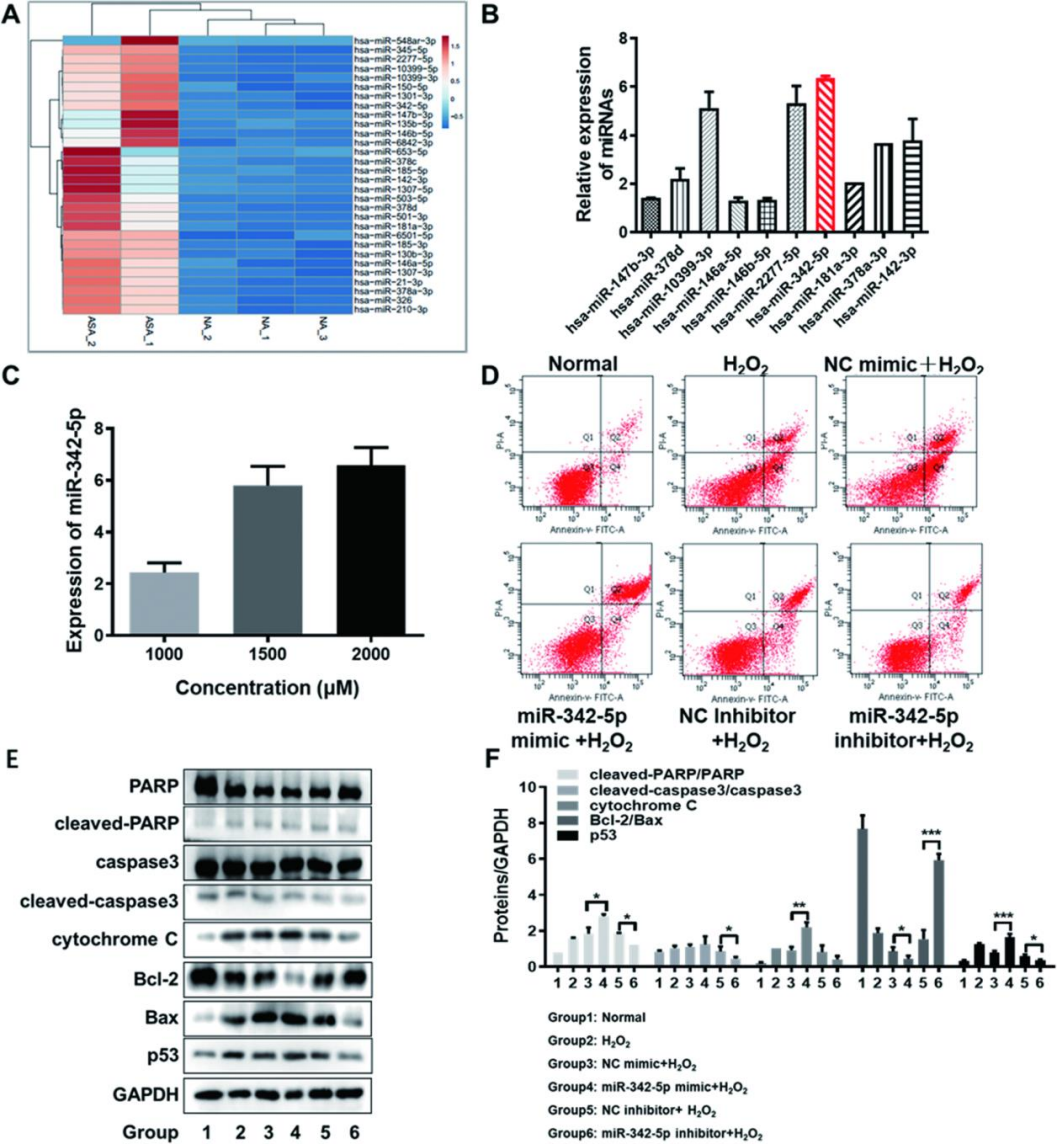
**The screened miR-342-5p facilitated the apoptosis of HUVECs impaired by H_2_O_2_.** (**A**) The heat map of top-30 upregulated miRNAs ranked by log2FC value in RNA-sequencing analysis was generated by the online tool ClustVis. (**B**) The expression levels of ten candidate miRNAs were determined in HUVECs impaired by H_2_O_2_ (1500 uM) by qRT-PCR assay. The relative expression of has-miR-342-5p achieved the highest among these ten miRNAs. (**C**) The expression of miR-342-5p was detected with the gradually increasing concentrations of H_2_O_2_ (1000, 1500 or 2000 uM) to treat HUVECs. Results indicated that miR-342-5p expression increased along with the increasing concentration of H_2_O_2_. (**D**) HUVECs were separately transfected with miR-342-5p mimic, inhibitor and their corresponding NC, followed by treating with 1500 uM of H_2_O_2_. Their apoptotic rates were then analyzed by flow cytometry assay. The apoptosis rate was increased in miR-342-5p mimic group (miR-342-5p mimic+H_2_O_2_) and decreased in miR-342-5p inhibitor group (miR-342-5p inhibitor+H_2_O_2_) when separately compared to their corresponding NC group. (**E**) HUVECs were separately transfected with miR-342-5p mimic, inhibitor and their corresponding NC, followed by treating with 1500 uM of H_2_O_2_. The well-known apoptin (PARP, caspase 3, cytochrome C, Bcl-2, Bax and p53) in treated HUVECs was evaluated by western blot assay. The expression levels of cleaved-PARP/PARP, cleaved-caspase3/caspase3, cytochrome C, and p53 were elevated while the ratio of Bcl-2 to Bax was declined in miR-342-5p mimic group (miR-342-5p mimic+H_2_O_2_). Opposite results were obtained in miR-342-5p inhibitor group (miR-342-5p inhibitor+H_2_O_2_). (**F**) The corresponding grey-scale maps of (**E**) were shown. *, *P*<0.05, **, *P*<0.01, ***, *P*<0.001. MiR-342-5p, microRNA-342-5p; HUVECs, human umbilical vein endothelial cells; FC, fold change; qRT-PCR, quantitative real-time polymerase chain reaction; NC, negative control; GAPDH, glyceraldehyde 3-phosphate dehydrogenase.

In order to further screen the aimed miRNA (s) associated with atherosclerosis, the expression profile of differentially expressed miRNAs obtained from RNA-sequencing was imported into the online tool ClustVis. Ranked by log2FC value, the top-30 upregulated miRNAs were picked out (Supplementary Table 1). The heat map of their expression profile was visualized in Figure 3A. Subsequently, ten miRNAs (namely miR-147b-3p, miR-378d, miR-10399-3p, miR-146a-5p, miR-146b-5p, miR-2277-5p, miR-342-5p, miR-181a-3p, miR-378a-3p and miR-142-3p) were considered as the candidates, who were top ranked in RNA-sequencing or reportedly associated with atherosclerosis. The expression levels of these ten candidate miRNAs were determined in HUVECs impaired by H_2_O_2_ (1500 uM) by qRT-PCR assay. As clearly illustrated in Figure 3B, the relative expression of has-miR-342-5p achieved the highest among these ten miRNAs in lesion model for HUVECs. The expression of miR-342-5p was thereupon detected with the gradually increasing concentrations of H_2_O_2_ (1000, 1500 or 2000 uM) to treat HUVECs. Obviously, this miRNA expression gradually fortified with the increasing concentration of H_2_O_2_ (Figure 3C), and the corresponding upregulated levels of miR-342-5p were 2.43-fold, 5.82-fold and 6.51-fold, respectively. MiR-342-5p was therefore selected as our aimed miRNA.

Next, the effect of miR-342-5p on the apoptosis of HUVECs’ lesion model was investigated. HUVECs were separately transfected with miR-342-5p mimic, inhibitor and their corresponding NC, followed by treating with 1500 uM of H_2_O_2_. The apoptosis and well-known apoptin (PARP, caspase 3, cytochrome C, Bcl-2, Bax and p53) of HUVECs were respectively evaluated by flow cytometry assay and western blot assay. As shown in Figure 3D and Table 2, the apoptosis rate in HUVECs transfected with miR-342-5p mimic group (miR-342-5p mimic+H_2_O_2_) was higher than that in NC group (NC mimic+ H_2_O_2_). Conversely, the apoptosis rate in HUVECs transfected with miR-342-5p inhibitor group (miR-342-5p inhibitor+H_2_O_2_) was lower than that in NC group (NC inhibitor + H_2_O_2_). These results indicated that miR-342-5p could promote the apoptosis of HUVECs. In terms of analysis of the well-known apoptin, results from western blot assay demonstrated that expression levels of cleaved-PARP/PARP, cleaved-caspase3/caspase3, cytochrome C, and p53 were elevated while the ratio of Bcl-2 to Bax was declined in HUVECs transfected with miR-342-5p mimic group (miR-342-5p mimic+H_2_O_2_) when compared to NC group (NC mimic+ H_2_O_2_), as shown in Figure 3E, 3F (*P*<0.05, *P*<0.01, *P*<0.001). The opposite results were obtained in HUVECs transfected with miR-342-5p inhibitor group (miR-342-5p inhibitor+H_2_O_2_) in Figure 3E, 3F (*P*<0.05, *P*<0.001). This revealed that miR-342-5p may promote the apoptosis of HUVECs mainly through mitochondrial-dependent apoptotic signaling pathway. Taken together, these findings unearth that miR-342-5p promotes the apoptosis of HUVECs impaired by H_2_O_2_.

**Table 2:** The effect of miR-342-5p on the apoptotic rate of HUVECs impaired by H_2_O_2_.

**Apoptotic rate (%)**	**Group**					
	**Normal**	**H_2_O_2_**	**NC mimic+H_2_O_2_**	**miR-342-5p mimic+H_2_O_2_**	**NC inhibitor+H_2_O_2_**	**miR-342-5p inhibitor+H_2_O_2_**
**Early apoptosis in Q_2_ stage**	2.4	6.5	8.7	23.1	12.1	7.9
**Middle and advanced apoptosis in Q_4_ stage**	2.5	14.4	17.9	7.4	10.0	7.8
**Total**	4.9	20.9	25.6	30.5	22.1	15.7

Abbreviations: miR-342-5p, microRNA-342-5p; HUVECs, human umbilical vein endothelial cells.

### PPP1R12B is identified as a target of miR-342-5p in HUVECs impaired by H_2_O_2_

**Figure 4:**
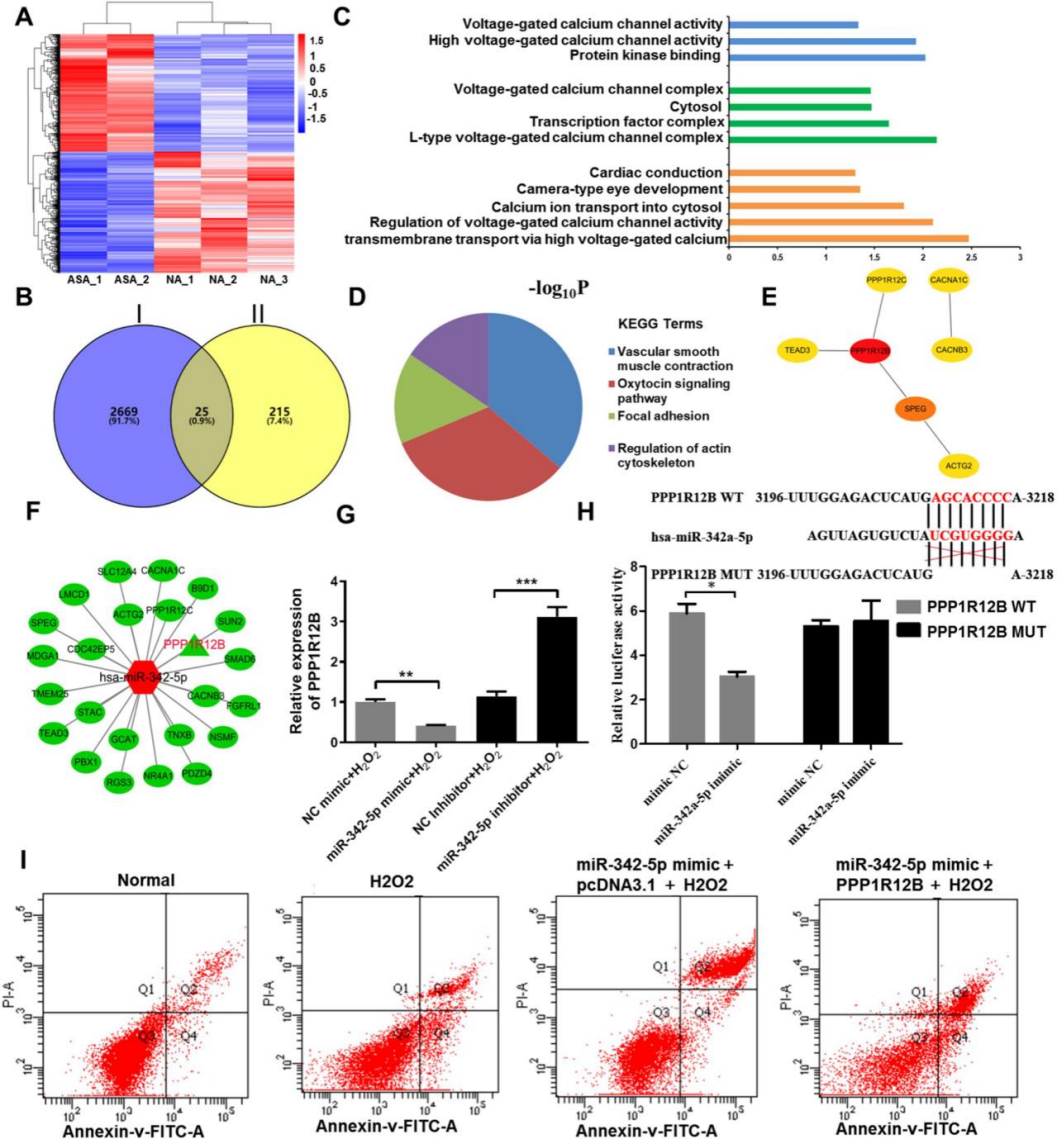
**PPP1R12B was identified as a target of miR-342-5p according to bioinformatics analyses and test verification.** (**A**) R cluster analysis of the differentially expressed mRNAs obtained from RNA-sequencing analysis. The red presents upregulated mRNAs and the blue indicates downregulated mRNAs. (**B**) The downregulated mRNAs in RNA-sequencing and the common genes from three databases (Miranda, PITA and RNAhybrid) were overlapped to obtain twenty-five genes. Circle I stands for the downregulated mRNAs in RNA-sequencing. Circle II represents the common genes obtained from Miranda, PITA and RNAhybrid databases. (**C**) GO terms and (**D**) KEGG signaling pathway enrichment analysis of twenty-five overlapped genes. (**E**) The hub genes extracted from twenty-five overlapped genes by Hubba plug-in according to degree score. (**F**) The network interaction between miR-342-5p and twenty-five overlapped genes was visualized by Cytoscape. The red shape node represents upregulated in atherosclerosis and the green shape node suggests downregulated in atherosclerosis. PPP1R12B as aimed mRNA is highlighted in red. (**G**) The effect of miR-342-5p on PPP1R12B expression in HUVECs’ lesion model was detected by qRT-PCR analysis. **, *P*<0.01, ***, *P*<0.001. (**H**) The target gene of miR-342a-5p, PPP1R12B, was identified by dual luciferase reporter gene assay. *, *P*<0.05. (**I**) HUVECs were co-transfected with miR-342-5p mimic and PPP1R12B overexpression vector, while pcDNA 3.1 was set as the control group, followed by treating with 1500 uM of H_2_O_2_. Their apoptotic rates were then analyzed by flow cytometry assay. miR-342-5p, microRNA-342-5p; mRNA, messenger RNA; GO, Gene ontology; KEGG, Kyoto Encyclopedia of Genes and Genomes; HUVECs, human umbilical vein endothelial cells; qRT-PCR, quantitative real-time polymerase chain reaction; NC, negative control.

In general, genes with the similar expression pattern could be gathered together according to Cluster analysis, suggesting that they possibly have common functions or participate in common metabolic and signaling pathways. The differentially expressed mRNAs obtained from RNA-sequencing analysis were used to conduct [R] Cluster analysis. This result was displayed in Figure 4A, among which the red presented upregulated mRNAs and the blue indicated downregulated mRNAs. In order to screen a target of miR-342-5p in atherosclerosis, three databases, Miranda, PITA and RNAhybrid, were initially employed for prediction, and 240 overlapped genes were thus obtained. Subsequently, as shown in Figure 4B, 2694 downregulated mRNAs in RNA-sequencing and these 240 genes obtained from three databases were further overlapped. Twenty-five genes were thus obtained, which were not only targets of miR-342-5p, but also downregulated in atherosclerosis. Next, these twenty-five genes were used for GO and KEGG enrichment analyses. As shown in Figure 4C and Table 3, GO analysis revealed that their biological processes were essentially enriched in calcium ion transmembrane transport via high voltage-gated calcium channel, regulation of voltage-gated calcium channel activity and calcium ion transport into cytosol. When it turned to cellular component, they were predominantly concentrated in L-type voltage-gated calcium channel complex, transcription factor complex and cytosol. As for molecular function, they were generally centralized on protein kinase binding, high voltage-gated calcium channel activity and voltage-gated calcium channel activity. As for KEGG analysis, results presented in Figure 4D and Table 4 clearly conveyed the information that these overlapped genes were predominately associated with vascular smooth muscle contraction and oxytocin signaling pathway, which have reportedly been involved in occurrence and development of atherosclerosis [23–27]. Furthermore, the interaction of these twenty-five overlapped genes was first analyzed by STRING, and then imported into Cytoscape. The hub genes were thus obtained in Figure 4E and Table 5 as analyzed by Hubba plug-in according to degree score. Among these hub genes, PPP1R12B with the highest degree score was simultaneously associated with vascular smooth muscle contraction and oxytocin signaling pathway as above KEGG analysis indicated. In addition, the network interaction between these twenty-five overlapped genes and miR-342-5p was visualized by Cytoscape as displayed in Figure 4F. In no small measure was therefore PPP1R12B a target of miR-342-5p in atherosclerosis. To verify this result, the effect of miR-342-5p on PPP1R12B expression in HUVECs’ lesion model was explored. As shown in Figure 4G, PPP1R12B expression exerted a decline in miR-342-5p overexpressing group (miR-342-5p mimic+H_2_O_2_) in comparison with NC group (NC mimic+ H_2_O_2_). As for miR-342-5p knockdown group, reverse results were obtained. These results indicated that miR-342-5p could constrain PPP1R12B expression in HUVECs’ lesion model. As shown in Figure 4H, the dual luciferase reporter gene assay confirmed that PPP1R12B was a target of miR-342a-5p. The relative luciferase activity was decreased in co-transfection of pGL3- PPP1R12B -WT with miR-342a-5p mimic, compared with the control of mimic NC (*P*< 0.05), and there was no significant difference in luciferase activity in co-transfection of pGL3- PPP1R12B -Mut with miR-342a-5p mimic (*P*> 0.05), indicating that miR-342a-5p was bound to the PPP1R12B gene. Additionally, miR-342-5p mimic and PPP1R12B overexpression vector were transfected into HUVEC, and pcDNA3.1 was set as the control group, followed by H2O2 induction at 1500uM. According to Figure 4I, the apoptosis rate of HUVEC was decreased after transfection of PPP1R12B overexpression vector compared with pcDNA3.1 group, indicating that PPP1R12B could reverse the apoptosis caused by miR-342-5p to some extent. Apoptosis rate of HUVECs at 1500uM of H_2_O_2_ in each group was presented in Table 6: 7.1% (Normal), 19.0% (H2O2), 29.3% (miR-342-p mimic+pcDNA3.1+H2O2) and 21.0% (miR-342-p mimic+PPP1R12B+H2O2), respectively. The evidence upon above sides unearth that PPP1R12B is a target of miR-342-5p in HUVECs impaired by H_2_O_2_.

**Table 3:** The enriched GO terms of twenty-five overlapped genes in atherosclerosis.

**Category**	**GO Term**	**Function description**	**Gene count**	**P value**	**Gene list**
Biological Process	GO:0061577	calcium ion transmembrane transport via high voltage-gated calcium channel	2	0.003	CACNB3, CACNA1C
	GO:1901385	regulation of voltage-gated calcium channel activity	2	0.008	STAC, CACNB3
	GO:0060402	calcium ion transport into cytosol	2	0.016	CACNB3, CACNA1C
	GO:0043010	camera-type eye development	2	0.044	B9D1, CACNA1C
	GO:0061337	cardiac conduction	2	0.049	CACNB3, CACNA1C
Cellular Component	GO:1990454	L-type voltage-gated calcium channel complex	2	0.007	CACNB3, CACNA1C
	GO:000566	transcription factor complex	3	0.022	SMAD6, NR4A1, PBX1
	GO:000582	cytosol	9	0.034	ACTG2, STAC, RGS3, B9D1, SMAD6, PPP1R12B, PIP5K1C, CACNB3, CDC42EP5
	GO:000589	voltage-gated calcium channel complex	2	0.034	CACNB3, CACNA1C
Molecular Function	GO:0019901	protein kinase binding	4	0.009	SLC12A4, PPP1R12B, PPP1R12C, CACNB3
	GO:0008331	high voltage-gated calcium channel activity	4	0.012	CACNB3, CACNA1C
	GO:0005245	voltage-gated calcium channel activity	4	0.046	CACNB3, CACNA1C

Abbreviations: GO, Gene ontology.

**Table 4:** The enriched signaling pathways for twenty-five overlapped genes in atherosclerosis.

**Category**	**KEGG Term**	**Function description**	**Gene count**	**P value**	**Gene list**
KEGG pathway	hsa04270	Vascular smooth muscle contraction	4	0.001	ACTG2, PPP1R12B, PPP1R12C, CACNA1C
	hsa04921	Oxytocin signaling pathway	4	0.002	PPP1R12B, PPP1R12C, CACNB3, CACNA1C
	hsa04510	Focal adhesion	3	0.048	TNXB, PPP1R12B, PPP1R12C
	hsa04810	Regulation of actin cytoskeleton	3	0.048	PPP1R12B, PPP1R12C, PIP5K1C

Abbreviations: KEGG, Kyoto Encyclopedia of Genes and Genomes.

**Table 5:** The hub genes ranked by score as analyzed by Hubba plug-in.

**Rank Number**	**Name**	**Score**
1	PPP1R12B	3.50
2	SPEG	3.00
3	TEAD3	2.33
4	PPP1R12AC	2.33
5	ACTG2	2.17
6	CACNB3	1.00
7	CACNA1C	1.00

**Table 6:** The apoptotic rates of HUVECs exposed to H2O2 in different groups.

**Apoptotic rate (100%)**	**Normal**	**H2O2**	**miR-342-p mimic+pcDNA3.1+H2O2**	**miR-342-p mimic+PPP1R12B+H2O2**
**Early apoptosis in Q_2_ stage**	4.6	8.4	23.3	12.7
**Middle and advanced apoptosis in Q_4_ stage**	2.5	10.6	6.0	8.3
**Total**	7.1	19.0	29.3	21.0

### ADSCs-derived exosomes inhibit the expression of miR-342-5p in HUVECs impaired by H_2_O_2_

**Figure 5:**
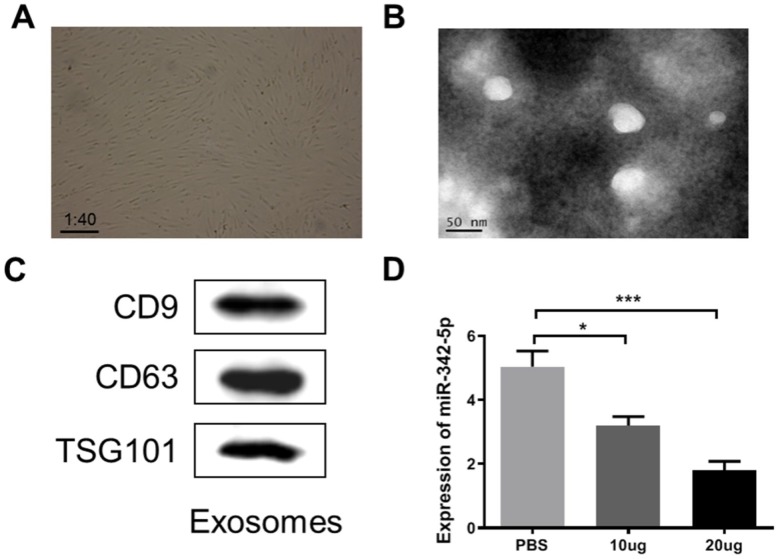
**ADSCs-derived exosome was characterized and constrained the expression of miR-342-5p in HUVECs impaired by H_2_O_2_.** (**A**) The spindle-like ADSCs were observed through the microscope at a magnification of ×40. (**B**) TEM analysis presented that exosomes were round membrane-bound vesicles with 30 to 150 nm diameter. Scale bar indicates 50 nm. (**C**) The common marker proteins for exosomes, CD9, CD63 and TGS101, were determined by western blot assay. (**D**) The expression level of miR-342-5p was subsequently detected by qRT-PCR assay after added the obtained exosomes at a mass of 10 ug or 20 ug into HUVECs impaired by H_2_O_2_ at a concentration of 1500 uM. *, *P*<0.05, ***, *P*<0.001. ADSCs, adipose-derived mesenchymal stem cells; miR-342-5p, microRNA-342-5p; HUVECs, human umbilical vein endothelial cells; qRT-PCR, quantitative real-time polymerase chain reaction; PBS, phosphate-buffered saline.

Commonly accepted, ADSCs can differentiate into cardiomyocytes or endothelial cells [28], and are extensively applied in clinical trials involved in regeneration of cardiac tissue, angiogenesis, and prevention of ischemia [29, 30]. Exosomes from ADSCs have been reported to be associated with angiogenesis, cardiac function and infarct size after ischemia [31]. It was therefore tempting to speculate that ADSCs-derived exosomes, to some extent, was participated in the development of atherosclerosis. With an aim to initially reveal the potential of ADSCs-derived exosomes involved in atherosclerosis, their effect on miR-324-5p was analyzed. At first, ADSCs were observed via a microscope at a magnification of ×40 after isolated and cultured for 24 h. As clearly shown in Figure 5A, a large proportion of the adherent cells were in spindle-like shape at the stage of cell culture. Next, exosomes purified from the culture supernatants of ADSCs were characterized by TEM. Results in Figure 5B displayed that exosomes were round membrane-bound vesicles with 30 to 150 nm diameter. As the common marker proteins for exosomes, expression levels of CD9, CD63 and TSG101 were determined by western blot assay. As expected, CD9, CD63 and TSG101 were detectable and presented in extracted exosomes (Figure 5C). Finally, the obtained exosomes at a mass of 10 ug or 20 ug was respectively added into HUVECs impaired by H_2_O_2_ at a concentration of 1500 uM. The expression level of miR-342-5p was subsequently detected by qRT-PCR assay. As shown in Figure 5D, miR-342-5p presented an evidently decreased expression level in exosomes group (both 10 ug and 20 ug) when compared to PBS normal group (*P*<0.05, *P*<0.001). Taken together, these findings suggest that ADSCs-derived exosomes inhibit the expression of miR-342-5p in HUVECs impaired by H_2_O_2_.

## DISCUSSION

In the present study, an atherosclerosis-associated miRNA, miR-342-5p, was screened by RNA- sequencing and bioinformatics analyses, and identified in favorably constructed lesion model for HUVECs. In particular, PPP1R12B was found to be a target of miR-342-5p in favorably constructed lesion model for HUVECs based on a series of bioinformatics analyses and verification tests. Given that ADSCs have strong differentiated capacity into endothelial cells and exosomes derived from them potentially play a vital role in angiogenesis [28–30], we fundamentally investigated the regulatory role of ADSCs-derived exosomes in miR-324-5p. Results indicated that the obtained ADSCs-derived exosomes could restrain the expression of miR-324-5p in lesion model for HUVECs.

As particularly a driving force in the initiation and development of atherosclerosis, damage or dysfunction of endothelial cells restrains platelet aggregation, leukocyte adhesion and the proliferation of vascular smooth muscle cells [32]. To realize the aim of studying the role of aimed miRNA in atherosclerosis in vitro, the lesion model for HUVECs was successfully constructed through the following experiments and verified tests: 1) The IC_50_ of H_2_O_2_ to impair HUVECs was first evaluated and calculated as 1538 uM; 2) Hoechst staining kit assay and flow cytometry assay conjointly demonstrated the increasing concentrations of H_2_O_2_ led to a considerable increase of apoptotic body or apoptosis rate in HUVECs; 3) More importantly, western blot assay indicated the enhanced concentrations of H_2_O_2_ contributed to the obvious arisen expression of cleaved-PARP/PRAP, cleaved-caspase3/caspase 3, cytochrome C, and p53, and the evident declined ratio of Bcl-2 to Bax.

Recently, in terms of atherogenesis, the theme of the regulatory roles of miRNAs in biological processes of endothelial cells has aroused great attention. For example, Sun, X. et al. point out that miRNA-181b participates in the anti-inflammatory phenotype of endothelial cells at athero-protected regions of arteries, probably through keeping NF-κB from translocating from the cytoplasm to the nucleus by targeting importin subunit α3 [33, 34]. Iaconetti, C. et al. and Daniel, J.M. et al. identify that miR-92a deteriorates endothelial repair via suppression of proliferation and migration of endothelial cells [35, 36]. Another example of this is the study in which miR-126-5p improves endothelial regeneration after denudation injury by targeting the mRNA encoding delta homologue 1 [21, 37]. Therefore, the aimed miRNA associated with atherosclerosis needed to be screened at first before this work moved towards investigation of mechanism. Our exploration to screen largely atherosclerosis-associated miRNA revealed encouraging results. At first, RNA-sequencing analysis with reliability demonstration (namely analysis of biological replicates) revealed 141 miRNAs differentially expressed in atherosclerosis samples, wherein 68 were upregulated and 73 were downregulated. Secondly, ten miRNAs (namely miR-147b-3p, miR-378d, miR-10399-3p, miR-146a-5p, miR-146b-5p, miR-2277-5p, miR-342-5p, miR-181a-3p, miR-378a-3p and miR-142-3p) were further screened with the criteria of top ranked in RNA-sequencing or reportedly associated with atherosclerosis. Results from qRT-PCR assay showed has-miR-342-5p achieved the highest relative expression in lesion model for HUVECs among these ten miRNAs. Finally, the gradually increasing concentrations of H_2_O_2_ contributed to fortify the expression of miR-342-5p. Furthermore, the promoted role of miR-342-5p in the apoptosis of HUVECs impaired by H_2_O_2_ was also evaluated and verified. MiR-342-5p was therefore selected as our aimed miRNA. In fact, later in searching reported literature, miR-342-5p has been identified as a potential biomarker for atherosclerosis, covering the aspects of inflammatory cytokines, inflammatory macrophage or injury [38–40]. However, these miR-342-5p-reported researches didn’t focus on its association with endothelial cells and the possible regulatory mechanism, which partially prompted us to conduct next analysis.

Accumulating evidence has demonstrated that miRNAs are remarkably stable in circulating blood [41] and generally regulate the post-transcription expression of target mRNAs [12]. According to RNA-sequencing analysis, prediction by databases, GO and KEGG enrichment analyses, we conducted the functional enrichment analysis of DEMs in both atherosclerosis and healthy people. It was found that the DEMs were mainly enriched in BP (calcium ion transmembrane transport via high voltage-gated calcium channel, regulation of voltage-gated calcium channel activity, calcium ion transport into cytosol), MF (protein kinase binding, high voltage-gated calcium channel activity, voltage-gated calcium channel activity) and CC (L-type voltage-gated calcium channel complex, transcription factor complex, cytosol). Importantly, they are mainly related to calcium channels. Through literature search, calcium channels are closely associated with vascular function and play an important role in the development of atherosclerosis [42]. In addition, studies have demonstrated that calcium channel blockers have an anti-atherosclerosis effect [43]. In the pathway enrichment analysis, these DEMs were mainly enriched in vascular smooth muscle contraction, oxytocin signaling pathway, etc. PPP1R12B was a target of miR-342-5p to a large extent, supported by its highest degree score and simultaneous association with vascular smooth muscle contraction and oxytocin signaling pathway. Notably, these two pathways have reportedly been involved in occurrence and development of atherosclerosis [23–27]. Later experiments concerning effect of miR-342-5p overexpression or knockdown on PPP1R12B expression further outlined that PPP1R12B was a target of miR-342-5p in HUVECs impaired by H_2_O_2_. Although little is known about the association of PPP1R12B with atherosclerosis according to existing reports, the finding we revealed provided a valuable cue or direction for further and deeper investigation.

**Figure 6:**
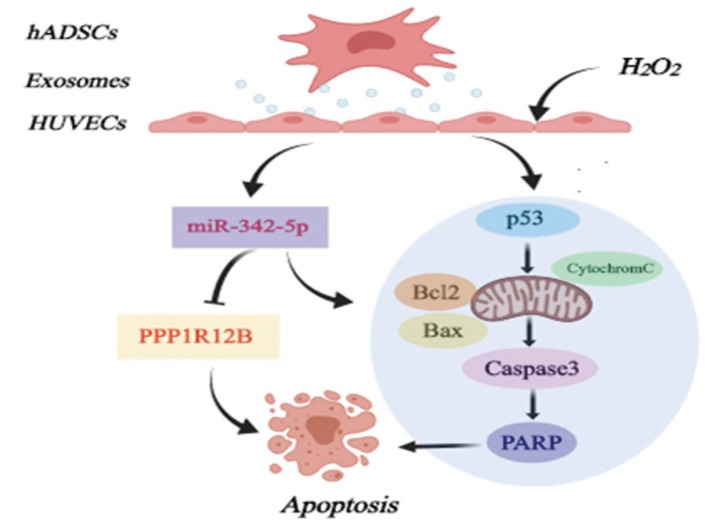
**Diagram of possible mechanism of miR-342-5p mediated by ADSCs-derived exosomes in lesion model for HUVECs to affect atherosclerosis.** hADSCs, human adipose-derived mesenchymal stem cells; miR-342-5p, microRNA-342-5p; HUVECs, human umbilical vein endothelial cells.

Exosomes are small membrane-bound vesicles and ADSCs-derived exosomes have been reported to possess cardioprotection supported by increased angiogenesis and cardiac function or other aspects [31, 44]. Recent studies have demonstrated that exosomes mediate several miRNAs which are potentially a key feature of acting on atherosclerosis, exemplified by miR-155 [45] and miR-223 [46]. In our work, ADSCs in spindle-like shape and thus derived exosomes were characterized. Interestingly, ADSCs-derived exosomes inhibited the expression of miR-342-5p in HUVECs impaired by H_2_O_2_. Importantly, as verified in our former tests, miR-342-5p could induce the apoptosis of HUVECs impaired by H_2_O_2_ probably through mitochondrial-dependent apoptotic signaling pathways_._ These results thus conjointly unearthed the possibility of exosomes of this kind regulating miR-342-5p to protect endothelial cells, thus combating atherosclerosis (Figure 6).

In conclusion, these results demonstrated a screened miR-342-5p associated with atherosclerosis. More than anything, this work preliminarily revealed a possible mechanism in which miR-342-5p mediated by ADSCs-derived exosomes could protect endothelial cells against atherosclerosis. These findings may reveal valuable insights into the role of miR-342-5p in the pathogenesis of atherosclerosis, and further indicate the potential of miR-342-5p as a novel therapeutic target for atherosclerosis. Despite these promising results as mentioned above, this study has some limitations that warrant further discussion. For example, dual luciferase reporter assays to verify PPP1R12B as a target of miR-342-5p is absent in this study. The regulatory mechanism between miR-342-5p and its screened target (PPP1R12B), or the role of ADSCs-derived exosomes in the former both (miR-342-5p and PPP1R12B) in atherosclerosis is not further focused on and explored. In addition, the associations of miR-342-5p and PPP1R12B as well as ADSCs-derived exosomes with mitochondrial-dependent apoptotic signaling pathway are also not investigated. Therefore, further study, taking all these aspects into account, will be warranted.

## MATERIALS AND METHODS

### Ethics statement

The specimens used in this study were collected from Central Laboratory of Liaocheng People’s Hospital with the participants’ written informed consent. This study was approved by the Ethics Committee of Liaocheng People’s Hospital.

### RNA-sequencing analysis

Three cases of atherosclerotic plaque collected from patients with atherosclerosis and two cases of normal carotid artery from patients with automobile accident were separately used for RNA-sequencing. Before RNA-sequencing, analysis of biological replicates was conducted by Novogene Bioinformatics Technology Co. Ltd (Beijing, China). The data about differentially expressed miRNA and mRNA in atherosclerosis samples were also obtained from Novogene Bioinformatics Technology Co. Ltd (Beijing, China).

### Visualization of differentially expressed miRNAs and mRNAs in atherosclerosis samples

The RNA-sequencing data were further employed to visualize differentially expressed miRNAs and mRNAs in atherosclerosis samples by a series of analytical tools as appropriate. In terms of the volcano plot, limma package [47] was applied to analyze differentially expressed miRNAs and mRNAs between Atherosclerosis and Healthy groups with the criteria of |log_2_FC|>1 and *P* < 0.05. Later, the results were visualized by Sanger Box. As for the heat map, the expression profile of differentially expressed miRNAs obtained from RNA-sequencing was imported into the online tool ClustVis (https://biit.cs.ut.ee/clustvis/). [R] Cluster analysis was used to evaluate the differentially expressed mRNAs obtained from RNA-sequencing analysis by Pheatmap package. With regards to Venn diagram, three databases, Miranda, PITA and RNAhybrid, were at first used for predicting targets for miR-342-5p. Afterwards, the intersection between overlapped genes in these three databases and the differentially expressed mRNAs obtained from RNA-sequencing analysis was visualized by online Venny website at https://bioinfogp.cnb.csic.es/tools/venny/index.html.

### Gene ontology (GO) and Kyoto Encyclopedia of Genes and Genomes (KEGG) enrichment analyses

The screened targets for miR-342-5p obtained from the intersecting Venn diagram were imported into online DAVID website at https://david.ncifcrf.gov/. Their GO and KEGG enrichment information was thus generated.

### Hub gene identified among the overlapped targets for miR-342-5p in Venn diagram

The screened targets for miR-342-5p obtained from the intersecting Venn diagram were imported into online STRING website at https://string-db.org/cgi/input.pl to obtain their interactions. Afterwards, the interactions were imported into Cytoscape and analyzed by Hubba plug-in dependent on degree score to get hub genes.

### Cell culture and transfection

HUVECs were purchased from the American Type Culture Collection (Manassas, VA, USA). HUVECs was cultured in DMEM supplemented with 10% fetal bovine serum, penicillin (100 U/mL), and streptomycin (100 mg/mL) at 37°C in a humidified atmosphere containing 5% CO_2_ (v/v). HUVECs were planted into 6-well plates at a density of 1×10^6^ cells/well. After cultured for 24 hours (h), HUVECs were separately transfected with miR-342-5p mimic, inhibitor or its corresponding negative control (NC), which were obtained from Ribobio (Guangzhou, China). Meanwhile, normal control was also designed. Subsequently, those transfected cells were treated with H_2_O_2_ at a concentration of 1500 uM for 24 h. Afterwards, cells were collected for the next assay.

### CCK-8 assay

In order to evaluate the optimal concentration of H_2_O_2_ to impair HUVECs, CCK-8 assay was performed in accordance with the manufacture’s protocol. In brief, HUVECs were seeded into 96-well plates at a density of 5000 cells/well. After incubation for 24 h, HUVECs were separately treated with varied concentrations of H_2_O_2_ (0, 500, 1000, 1500, and 2000 uM). Next, cells were added with 10 μL of CCK-8 reagent and then cultured for one day. The optical density (OD) value of each well was measured using the Multiskan FC (Thermo Fisher Scientific, Inc., Waltham, MA, USA) at 450 nm wavelength. The IC_50_ value of H_2_O_2_ to impair HUVECs was calculated using Graphpad software

### Hoechst staining kit assay

HUVECs were planted into 6-well plates at a density of 1×10^6^ cells/well, and then treated with H_2_O_2_ at varied concentrations of 0, 1000, 1500, and 2000 uM. After culture for 24 h, the clear supernatant extract was supplemented with 1 mg/mL of Hoechst 33342 kit (Beyotime Biotechnology, Shanghai, China) and then incubated in the dark for half an hour. Nuclei were observed using a fluorescence microscope.

### Flow cytometry assay

To evaluate the effect of concentrations of H_2_O_2_ on cell apoptosis of HUVECs, flow cytometry assay were performed using Annexin V-FITC/PI apoptosis detection kit (Beyotime Biotechnology, Shanghai, China) following the manufacturer’s instructions. In concrete, HUVECs were seeded into 6-well plates and treated with H_2_O_2_ at varied concentrations of 0, 1000, 1500, and 2000 uM. After centrifuged, the HUVECs were collected for apoptosis analysis, followed by being washed with ice-cold phosphate-buffered saline (PBS) and then stained with (FITC)-Annexin V and propidium iodide (PI). Finally, the apoptosis rate of HUVECs was measured and analyzed by BD FACSCalibur (BD, USA).

### Dual luciferase reporter assay

The dual luciferase reporter vector of PPP1R12B and the mutants of binding sites of PPP1R12B to the miR-342-5p were designed separately: pGL3- PPP1R12B -wild type (Wt) and pGL3 - PPP1R12B -mutation (Mut). We missed a sequence of binding bases in mutation vector. The two reporter plasmids were co-transfected into HUVEC with the plasmid that had overexpressed miR-342-5p and pRL-TK (internal reference plasmid expressing Renilla luciferase). After a 24 h transfection, a dual luciferase reporter system (Dual-Luciferase® Reporter Assay System, E1910, Promega, Madison, WI, USA) was adopted to determine luciferase activity, which was represented by the ratio of firefly luciferase to Renilla luciferase. The experiment was conducted in triplicates.

### RNA isolation and quantitative real-time polymerase chain reaction (qRT-PCR) assay

In accordance with the manufacturer’s instructions, the total RNA was extracted from the cells using Trizol reagent (Thermo Fisher Scientific; USA). In terms of mRNAs, the First-strand cDNA was synthesized from 1 μg of total RNA using SuperScript III reverse transcriptase (Invitrogen; USA) with oligo (dT) 20 primer. As for miRNAs, its reverse transcription was performed by using miDETECT A Track ^TM^ miRNA qPT-PCR Starter Kit (RIBOBIO, China). A AriaMx Real-Time PCR (Agilent Technologies; USA) using the SYBR Green Real-time PCR Master Mix (TOYOBO, Japan) was employed to conduct qRT-PCR assay. Comparative quantification was determined using the 2^−ΔΔCt^ method. U6 was used as an internal reference for miRNAs, and glyceraldehyde 3-phosphate dehydrogenase (GAPDH) was for mRNAs. The experiments were performed in triplicate. The primer sequences used in this study were summarized in Table 7.

**Table 7:** List of primers used in this study.

**Name**	**Sequence 5′-3′**
hsa-miR-147b-3p	GTGTGCGGAAATGCTTCTGCT
hsa-miR-378a-3p	ACTGGACTTGGAGTCAGAAGGC
hsa-miR-142-3p	TGTAGTGTTTCCTACTTTATGGA
has-miR-378d	ACUGGACUUGGAGUCAGAAA
hsa-miR-10399-3p	CTCTCGGACAAGCTGTAGGTC
hsa-miR-146a-5p	TGAGAACTGAATTCCATGGGTT
hsa-miR-146b-5p	TGAGAACTGAATTCCATAGGCTG
hsa-miR-2277-5p	AGCGCGGGCTGAGCGCTGCCAGTC
hsa-miR-342-5p	AGGGGTGCTATCTGTGATTGA
hsa-miR-181a-3p	ACCATCGACCGTTGATTGTACC
Universal primer	GCGAGCACAGAATTAATACGAC
U6-F	CTCGCTTCGGCAGCACA
U6-R	AACGCTTCACGAATTTGCGT
PPP1R12B-F	TCTTCTGCTAGGAGGTTCTCTTCT
PPP1R12B-R	CATACCCTCCCAAACTGCACC
GAPDH-F	ACGGATTTGGTCGTATTGGGCG
GAPDH-R	GCTCCTGGAAGATGGTGATGGG

Abbreviations: miRNA, microRNA; GAPDH, glyceraldehyde 3-phosphate dehydrogenase.

### The extraction and culture of ADSCs

Human facial adipose tissues were obtained from the Central Laboratory of Liaocheng People’s Hospital (Liaocheng, China). They were digested by collagenase type I for 2 h and DME/F12 complete media was used to terminate this digestion. Subsequently, this digested sample was centrifuged at 1000 rpm/min for 5 min to obtain cell-debris pellet. Normally, a small amount of ADSCs was observed after one week. Longer in one month, a large amount was observed. Their images of representative fields were visualized by microscope (Olympus Corporation, Tokyo, Japan) at a magnification of ×40.

### The isolation and characterization of ADSCs-derived exosomes

Using exosome isolation reagent (RiboBio, Guangzhou, China), exosomes were extracted from ADSCs’ supernatants in the absence of cell debris according to the manufacturer's guidelines. Final obtained exosomes were stored at -80°C to reserve for the following study. Meanwhile, the morphology of the isolated exosomes was monitored and characterized by means of transmission electron microscopy (TEM, JEM2010-HT, Tokyo, Japan). The diameter of exosomes was quantified by micrographs. Finally, the common marker proteins for exosomes, CD9, CD63 and TSG101, were detected by western blot assay.

### Effect of ADSCs-derived exosomes on miR-342-5p expression

The protein concentration of ADSCs-derived exosomes was 79.25ug/mL, which was measured by BCA Protein Assay Kit (Beyotime Biotechnology, Shanghai, China) following the manufacturer’s instructions. HUVECs (1 × 10^6^ cells in 6-well plates) were pre-cultured for 24 h. Afterwards, 1500 uM of H_2_O_2_ and a mass of ADSCs-derived exosomes (10 or 20 ug) were simultaneously added into HUVECs. An equivalent volume of exosome diluent PBS was added as the control group. After 24 h, the total RNA was extracted for qRT-PCR assay to detect the expression of miR-342-5p. The experiment was repeated at least three times independently.

### Western blot assay

Cells were lysed with lysis buffer (Beyotime Biotechnology, Shanghai, China) in the presence of the protease inhibitors (Roche, Basel, Switzerland) to extract total protein. Protein samples were electrophoresed on 12% SDS-PAGE gel, and then transferred to polyvinylidene difluoride (PVDF) membranes (Millipore, Bedford, MA, USA). After blocked in 1 × tris-buffered saline containing 0.1% Tween-20 (TBST) with 5% skim milk for 1 h, PVDF membranes were incubated with a 1:1000 dilution of different primary antibodies (CD9, CD63, TSG101, PARP, caspase3, cytochrome C, Bcl-2, Bax, p53, GAPDH, ABclonal) at 4°C overnight. Afterwards, these membranes were incubated with the corresponding secondary antibody (goat anti-mouse/anti-rabbit, 1:10000, ABclonal), which were labeled with horseradish peroxidase (HRP) at 37°C for 1 h. GAPDH was used as the internal control. An enhanced ECL kit (Pierce, Rockford, IL, USA) was employed to visualize signals. The images of the gels were scanned using Bio-Rad Gel Doc XR+ system (Bio-Rad, Hercules, CA, USA). Finally, the grey-scale maps of bands obtained were analyzed by image J.

### Statistics

The data were expressed as the mean ± standard deviation. Differences between two groups were analyzed using Student’s *t*-test. Statistical analyses were performed with GraphPad Prism software version 8.0 (La Jolla, CA, USA). The differences were considered to be statistically significant as a result of *P* < 0.05.

## Supplementary Material

Supplementary Table 1
