# Supplementary material for: Adipose-derived mesenchymal stem cells-derived exosome-mediated microRNA-342-5p protects endothelial cells against atherosclerosis
Source: Aging (Albany NY). 2020 Feb 24;12(4):3880–98. doi: 10.18632/aging.102857 (PMC7066923; doi:10.18632/aging.102857)
Supplement: Supplementary Table 1 [file aging-12-102857-s001..pdf]

SUPPLEMENTARY TABLE

Supplementary Table 1. The top-30 miRNAs upregulated in atherosclerosis according to log_2FC value.

NO.	miRNA	Samples				
		ASA_1	ASA_2	NA_1	NA_2	NA_3
1	hsa-miR-147b-3p	45.96	15.70	0.09	0.72	0.59
2	hsa-miR-378a-3p	1929.63	2411.42	114.52	187.96	86.26
3	hsa-miR-378d	82.94	132.12	2.91	9.85	2.97
4	hsa-miR-378c	141.75	317.88	5.82	20.31	6.78
5	hsa-miR-142-3p	371.20	1062.47	9.57	53.96	5.24
6	hsa-miR-326	12.50	16.39	0.19	1.32	0.48
7	hsa-miR-210-3p	94.91	120.39	1.78	11.06	5.47
8	hsa-miR-503-5p	4.40	8.11	0.47	0.36	0.24
9	hsa-miR-10399-3p	6.87	5.00	0.09	0.24	0.59
10	hsa-miR-548ar-3p	126.26	3.28	1.88	3.00	1.90
11	hsa-miR-146a-5p	11331.94	15325.74	557.93	2131.01	571.95
12	hsa-miR-1307-5p	4.93	14.14	0.66	1.08	0.36
13	hsa-miR-146b-5p	22257.62	12248.21	1803.99	2008.55	1433.63
14	hsa-miR-2277-5p	3.87	3.28	0.19	0.36	0.24
15	hsa-miR-1301-3p	23.42	16.73	2.44	2.52	0.83
16	hsa-miR-21-3p	946.85	1277.37	104.85	151.18	116.13
17	hsa-miR-653-5p	2.47	12.42	0.28	0.36	0.71
18	hsa-miR-185-3p	23.60	25.70	3.10	3.37	1.55
19	hsa-miR-1307-3p	128.20	167.82	18.68	22.83	8.92
20	hsa-miR-130b-3p	4.23	4.66	0.56	0.48	0.24
21	hsa-miR-342-5p	15.67	12.07	1.60	2.28	0.83
22	hsa-miR-345-5p	46.14	42.77	4.88	7.09	4.40
23	hsa-miR-501-3p	22.36	36.05	3.66	3.73	3.09
24	hsa-miR-181a-3p	31.34	51.57	4.22	6.73	4.52
25	hsa-miR-10399-5p	19.37	16.39	2.07	2.64	2.02
26	hsa-miR-185-5p	292.31	758.39	79.50	64.18	46.28
27	hsa-miR-6501-5p	2.82	2.93	0.09	0.12	0.48
28	hsa-miR-150-5p	221.70	159.71	13.42	40.62	18.68
29	hsa-miR-135b-5p	8.45	3.10	0.94	0.48	0.48
30	hsa-miR-6842-3p	30.29	18.63	4.13	2.28	3.21

Abbreviations: miRNA, microRNA; FC, fold change.
